# Stability and degradation in triple cation and methyl ammonium lead iodide perovskite solar cells mediated via Au and Ag electrodes

**DOI:** 10.1038/s41598-022-19541-6

**Published:** 2022-11-03

**Authors:** Kakaraparthi Kranthiraja, Mritunjaya Parashar, Ravindra K. Mehta, Sujan Aryal, Mahdi Temsal, Anupama B. Kaul

**Affiliations:** 1 Department of Electrical Engineering, PACCAR Technology Institute, University of North Texas, Denton, TX 76207 USA; 2 Department of Materials Science and Engineering, University of North Texas, Denton, TX 76207 USA

**Keywords:** Solar cells, Electrical and electronic engineering

## Abstract

Perovskite solar cells (PSCs), particularly based on the methyl ammonium lead iodide (MAPbI_3_) formulation, have been of intense interest for the past decade within the photovoltaics (PV) community, given the stupendous rise in power conversion efficiencies (PCEs) attributed to these perovskite formulations, where PCEs have exceeded 25%. However, their long-term stability under operational conditions and environmental storage are still prime challenges to be overcome towards their commercialization. Although studies on the intrinsic perovskite absorber stability have been conducted previously, there are no clear mechanisms for the interaction of electrode-induced absorber degradation pathways, which is the focus of this study. In this report, we have conducted a comprehensive analysis on the impact of the electrode collector layer, specifically Ag and Au, on the degradation mechanism associated with the MAPbI_3_ and a triple cation absorber, Cs_0.05_FA_0.79_MA_0.16_PbI_2.45_Br_0.55_. Notably, Au-based PSCs for both absorbers in an n-i-p architecture showed superior PCE over Ag-based PSCs, where the optimized PCE of MAPbI_3_ and triple cation-based PSCs was 15.39% and 18.21%, respectively. On the other hand, optimized PCE of MAPbI_3_ and triple cation-based PSCs with Ag electrodes was 3.02% and 16.44%, respectively. In addition, the Ag-based PSCs showed a rapid decrease in PCE over Au-based PSCs through operational stability measurements. We hypothesize the mechanism of degradation, arising from the Ag interaction with the absorber through the formation of AgI in the PSCs, leads to corrosion of the perovskite absorber, as opposed to the benign AuI when Au electrodes are used in the solar cell stack. Additionally, novel use of photoluminescence spectroscopy (PL) here, allowed us to access key features of the perovskite absorber in situ, while it was in contact with the various layers within the n-i-p solar cell stack. A quenching in the PL peak in the case of Ag-contacted MAPbI_3_ provided direct evidence of the Ag corrupting the optical properties of the absorber through the formation of AgI which our X-ray diffraction (XRD) results confirmed. This was supported by the fact that an emission peak was still present in the triple cation Ag-device. For the Au-contacted MAPbI_3_ the presence of a well-defined PL peak, though attenuated from the triple cation Au-device, suggested the AuI does not quell the emission spectrum for either the triple cation or the MAPbI_3_ absorber. The findings should aid in the understanding and design of new electrode materials with PSCs, which will help accelerate their introduction into the commercial sector in the future.

## Introduction

Perovskites are well known for their superb optical and optoelectronics transport properties since their discovery less than decade ago for photovoltaics (PV) applications^1–3^. As a result, perovskite solar cells (PSCs) have seen intense activity with power conversion efficiencies (PCEs) for single junction cells rising to 25.6% in less than one decade from just a few percent^4^. Despite the stupendous rise in PCEs that perovskites have enabled, the vulnerability of the methyl ammonium lead iodide MAPbI_3_, one of the earliest and most studied formulations for PSCs, to moisture, elevated temperatures and ultra-violet (UV) radiation, are major impediments towards commercializing PSCs as a viable PV technology^5,6^. The high sensitivity to environmental conditions in the ambient stems largely from the organic MA cation in the PSCs, given its hydrophilic nature, where photocurrent degradation via extended light soaking is also evident through its structural and thermal instability^7^. Numerous approaches have been used to ameliorate the stability of the MAPbI_3_ absorber, as well as the contact and interface layers, which also lead to degradation pathways within the solar cell stack^8^.

To comment more on the attributes of the absorber and its intrinsic structural and thermal stability, the *ABX*_*3*_ configuration of the perovskite structure is invoked. Here, ‘*A*’ denotes a large organic/inorganic cation (e.g., methylammonium (MA), formamidinium (FA), Cs^+^), *B* denotes an inorganic metal cation (e.g., Pb^2+^, Sn^2+^), and *X* denotes a halide anion (e.g., Cl^−^, Br^−^, I^−^). The structural stability of the perovskite structure can be determined by the Goldschmidt’s tolerance factor *t* which is defined as $t=\frac{{(r}_{A}+{r}_{X})}{\sqrt{2} ({r}_{B}+{r}_{X})}$, where *r*_*A*_, *r*_*B*_, and *r*_*X*_ are the effective ionic radii of the *A*, *B*, and *X* ions^9^. The ideal perovskite structure is formed when *t* = 1, but a feasible range of values for the formability of alkali metal halide perovskites is 0.813 < t < 1.107^10^. The usage of perovskites with mixed cations and halides has become essential to enhance stability since pure perovskite compounds suited for PV applications, notably MAPbX_3_, FAPbX_3_, and CsPbX_3_ (X = Cl, Br or I), have several drawbacks. The thermal instability of MAPbI_3_ is largely driven by the structural phase transition it experiences at temperatures as low as 55 °C, while contact with moisture due to its hydrophilic nature and exposure to light induces trap-states, leading to further degradation^11–14^. The FA cation has a slightly larger ionic radius of ~ 2.79 Å compared to the MA cation, yielding *t* ~ 0.987 for FAPbI_3_ and making it more cubic in contrast to MAPbI_3_^15^. However, the FAPbI_3_ perovskite exists in two different phases i.e. the cubic photoactive phase (α-FAPbI_3_) and the hexagonal non-photoactive phase (δ-FAPbI_3_). Owing to the greater size and asymmetric nature of the FA groups, the FAPbI_3_ perovskite suffers from a critical issue due to its phase instability, which results in a transition from the α-FAPbI_3_ phase to the δ-FAPbI_3_ phase at room temperature, aided by its sensitivity to solvents or humidity^16,17^. Alternatively, the purely inorganic CsPbI_3_ perovskite exhibits excellent thermal stability with a suitable band gap of ~ 1.73 eV^18^, but CsPbI_3_ crystallizes in a non-photoactive, orthorhombic δ-phase (“yellow” phase) at room temperature, while the cubic photoactive α-phase (“black” phase) is only stable at temperatures above 300 °C^18^.

As a result, pure perovskite compounds fall short, owing to thermal or structural instability. Therefore, mixing cations and halides has become an essential design approach in order to produce perovskite compounds with enhanced thermal and structural stability. Various mixed cation and mixed halide structures have been reported which show PCE > 20% with good reproducibility^19,20^. The recent success of MA/FA mixed perovskites shows that a little quantity of MA is already enough to promote preferred crystallization into the photoactive phase of the FA perovskite, resulting in a more thermally and structurally stable composition compared to pure MA or FA compounds^21^. This demonstrates how the MA may be viewed as a “crystallizer” (or stabilizer) of the black phase FA perovskite. As a result, it was inferred that the use of smaller cations, such as MA, is important in the development of the structurally stable black phase FA perovskite. Even with MA, it is difficult to ensure the FA perovskite with no residues of the yellow phase, which surprisingly is found even in solar cells exhibiting high PCE^17,22^. These yellow phase impurities must be avoided since even tiny amounts impact the crystal development and shape of the perovskite, preventing effective charge collection and reducing device performance. Just as the MA/FA double cation perovskite structures, several studies have shown the addition of a Cs cation (ionic radii ~ 1.81 Å) as a promising candidate to stabilize the Cs/MA and Cs/FA perovskite structures^21^. Due to the greater size difference between the Cs and FA, it is obvious that Cs is highly effective in pushing FA into the δ-perovskite phase. For MA on the other hand, crystallization of FA perovskite results, albeit at a considerably slower pace (since MA is only slightly smaller than FA), allowing a substantial percentage of the yellow phase to survive^21^. As previously stated, such MA/FA compounds already exhibit outstanding PCEs, and therefore their further improvement in stability is touted as an important route toward the commercialization of PSCs.

While materials engineering approaches have been used to enhance the structural and thermal stability of the intrinsic photoactive absorber material itself, through triple cation and mixed halide compositions as discussed above, other avenues for stability involving the entire device structure^22^, through encapsulation approaches have also been considered^8^. However, this approach is not effective to prevent internal material degradations pathways through interfaces^23^, ion diffusion^24^, and hysteresis^25^.

In the present work, we have conducted a comprehensive study of one feature within the solar cell stack, namely the electrode layer, to elucidate the degradation pathways prevalent in the more intrinsically stable triple cation, mixed halide absorber, specifically Cs_0.05_FA_0.79_MA_0.16_PbI_2.45_Br_0.55_. Besides the Cs_0.05_FA_0.79_MA_0.16_PbI_2.45_Br_0.55_ (triple cation) absorber, MAPbI_3_ was used as a reference in our tests, to further pin-point the degradation pathway mechanism mediated via the Anode electrode collector layer. The typical choices for the electrode materials with adequate band alignment to the underlying hole transport layer (HTL) in the n-i-p architecture are Al, Ag and Au. Metals such as Al and Ag often show detrimental effects on PSCs stability through the formation of respective oxides and iodides, i.e. AlOx and AgI^26,27^, but it is not clear to what extent the absorber has a role to play in this degradation pathway. On the other hand, Au is considered to be more stable but even so, recent reports demonstrate the migration of Au through the spiro-OMeTAD and into the perovskite layer under illumination^28^.

Here, we fabricated triple cation and MAPbI_3_ based n-i-p PSCs with both Ag and Au electrodes, where the Au-based PSCs showed higher PCE than the Ag-based PSCs. We attribute this difference to the rapid generation of AgI in Ag-based PSCs, over AuI in Au-based PSCs. The degradation mechanisms were elucidated through various material characterization probes conducted using X-ray diffraction (XRD), scanning electron microscopy (SEM) and photoluminescence (PL) spectroscopy to help shed insights on the electrode-mediated degradation mechanisms in the Ag- and Au-based PSCs. A novel use of PL here allowed us to access key features of the perovskite absorber in situ, while it was in contact with the various layers within the n-i-p solar cell stack. The laser used for the incoming excitation penetrated through the Spiro-OMeTAD layer and quenching in the PL intensity in the case of Ag-contacted MAPbI_3_ provided direct evidence of the Ag corrupting the optical properties of the absorber through the formation of AgI. On the other hand, an emission peak was still present in the triple cation Ag-devices. For the Au-contacted MAPbI_3_, the PL peak was still present, though attenuated from the triple cation Au-device, which suggested the AuI does not quell the emission spectrum for either the triple cation or the MAPbI_3_ absorbers. The incorporation of multiple cations, anions and halides have led PSCs towards higher stability and efficiency compared to pure archetypical perovskites, which are typically comprised of MAPbX_3_, FAPbX_3_ and CSPbX_3_ (X = Br or I). Using FAPbI_3_ to form mixed cation/halide perovskites rather than pure MAPbI_3_ helps in reducing the optical band gap slightly; for example, the band gap *E*_*g*_ of FAPbI_3_ is ~ 1.502 eV while that of MAPbI_3_ is ~ 1.563 eV^29^. The reduced, slightly more favorably tuned bandgap of the former, coupled with its higher environmental stability, thus leads to an increment in efficiency^21^, we observe here with the Au electrodes.

## Results and discussion

### Photovoltaic properties of Ag and Au-based PSCs

**Figure 1:**
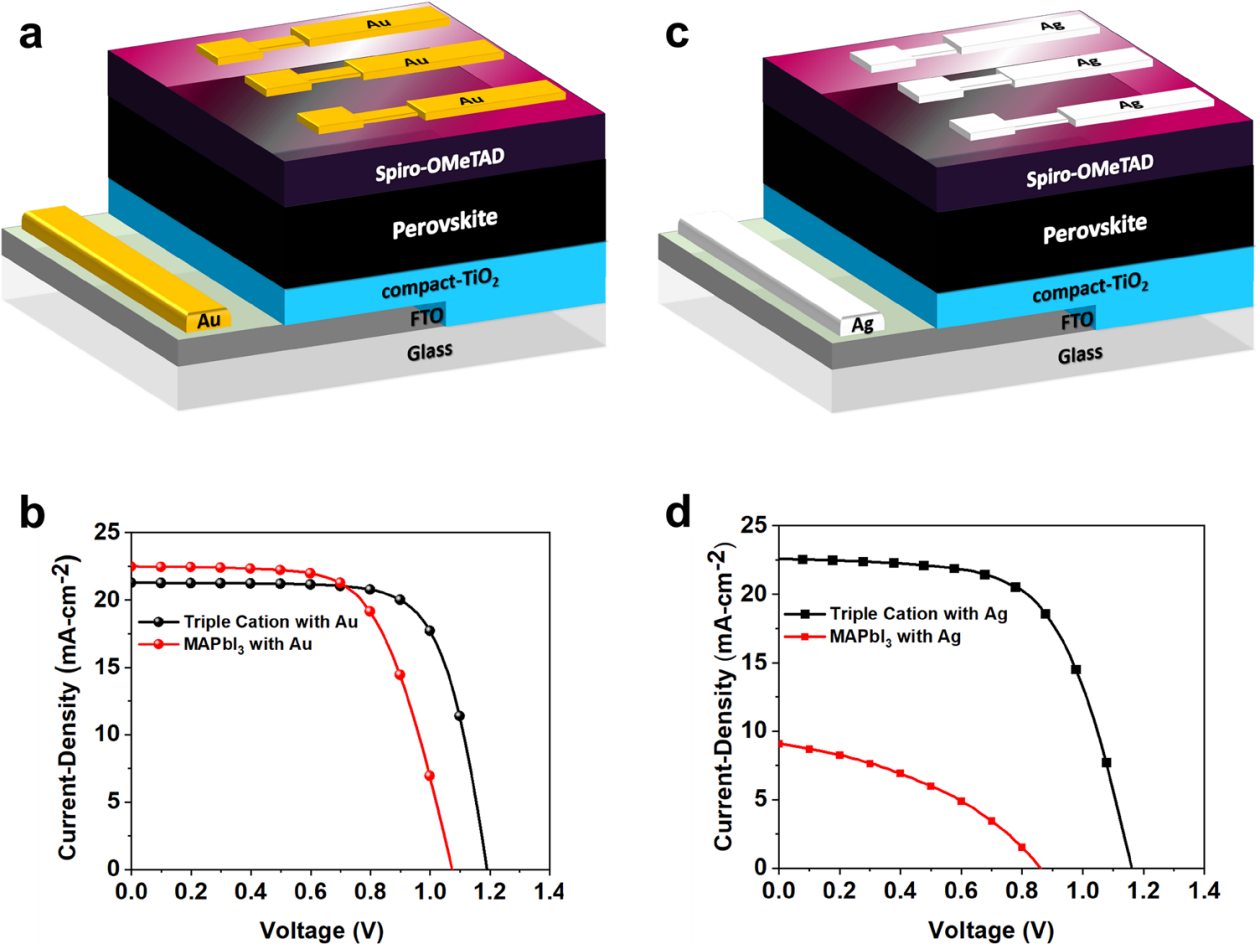
(**a**) Device architecture of n-i-p (FTO/compact-TIO_2_/triple cation or MAPbI_3_ perovskite/Spiro-OMeTAD/Au) PSC and, (**b**) optimized *J–V* Characteristics with Au-electrodes. (**c**) Device architecture of n-i-p (FTO/compact-TIO_2_/triple caiton or MAPbI_3_ perovskite/Spiro-OMeTAD/Ag) PSC and, (**d**) optimized *J–V* characteristics with Ag-electrodes.

The details of the absorber material synthesis and PSC device fabrication process are provided in “Materials and methods” section. In this work, we formed PSCs based on the planar n-i-p architecture without the use of the mesoporous layer, where the use of the mesoporous layer to enhance stability is incidentally, still under debate^30^. Figure 1A depicts the triple cation and MAPbI_3_ absorber devices contacted with Au, comprising of FTO/c-TiO_2_/perovskite (triple cation or MAPbI_3_)/spiro-OMeTAD/Au. The corresponding *J-V* Characteristics are shown in Fig. 1b. In Fig. 1c, the Ag-electrode contacted devices are shown for both absorbers with the following layer stacking: FTO/c-TiO_2_/perovskite (triple cation or MAPbI_3_)/spiro-OMeTAD/Ag, where the corresponding *J–V* Characteristics are illustrated in Fig. 1d for the two absorbers.

The maximum PCE of the triple cation and MAPbI_3_ based PSCs arose with the use of Au-electrodes shown in Fig. 1a, where the PCEs obtained are 18.21% (*V*_*oc*_ = 1.19 V, *J*_*sc*_ = 21.29 mA cm^−2^ and FF = 71.87%) for the triple cation, and 15.39% (*Voc* = 1.01 V, *Jsc* = 22.05 mA cm^−2^ and FF = 60.20%) for the MAPbI_3_. In a similar fashion, the maximum PCE of triple cation and MAPbI_3_ based PSCs contacted with Ag-collector electrodes are 16.44% (*V*_*oc*_ = 1.16 V, *J*_*sc*_ = 22.59 mA cm^−2^ and FF = 62.67%) and 3.02% (*V*_*oc*_ = 0.85 V, *J*_*sc*_ = 9.11 mA cm^−2^ and FF = 38.63%), respectively. The optimized PV parameters are summarized in Table 1. It is interesting to note that both triple cation and MAPbI_3_ based PSCs with Au showed enhanced PCE over the Ag counterparts. The high PCE of Au-based PSCs maybe attributable to the efficient charge extraction and better stability of the Au compared to the Ag electrode, which we will further gauge through our material characterization studies discussed later in this work. The low PCE of Ag-based PSCs is likely to do with the immediate degradation of the MAPbI_3_ based PSCs and rapid formation of AgI^31^. In addition, triple cation-based PSCs with Ag showed relatively better PCE due to the more intrinsic stability of the triple cation absorber itself, over the MAPbI_3_. However, though the triple cation absorber is intrinsically more stable than MAPbI_3_ through the incorporation of Cs^+^ and Br^−^, it still suffers from metal-induced degradation^32^. Hence, triple cation-Ag PSC showed a lower efficiency compared to Au-based devices.

**Table 1:** Optimized photovoltaic parameters of n-i-p PSCs based on MAPbI_3_ and triple cation as absorbers contacted with Au and Ag collector electrodes.

PSC	*V*_*oc*_ (V)	*J*_*sc*_ (mA.cm^−2^)	FF (%)	PCE (%)	*R*_sh_ (kΩ-cm^2^)	*R*_s_ (Ω-cm^2^)
Triple-Au	1.19	21.29	71.87	18.21	5456.34	6.75
MAPbI_3_-Au	1.01	22.05	60.20	15.39	2090.35	10.54
Triple-Ag	1.16	22.59	62.67	16.44	1995.67	10.11
MAPbI_3_-Ag	0.85	9.11	38.63	3.02	251.20	31.69

### Time-dependent and recombination analysis for Au-contacted electrodes

Next, we performed time-dependent measurements of PCE for triple cation and MAPbI_3_ based PSCs with Au electrodes, since Ag-contacted devices prevented us from gathering the equivalent data due to their instability and unmeasurable PCE upon first illumination under the solar simulator. The origin of this may have to do with the rapid reaction of iodine given its rapid migration and subsequent formation of AgI, as alluded to previously; this is confirmed through our subsequent material characterization studies presented later^31,33^.

**Figure 2:**
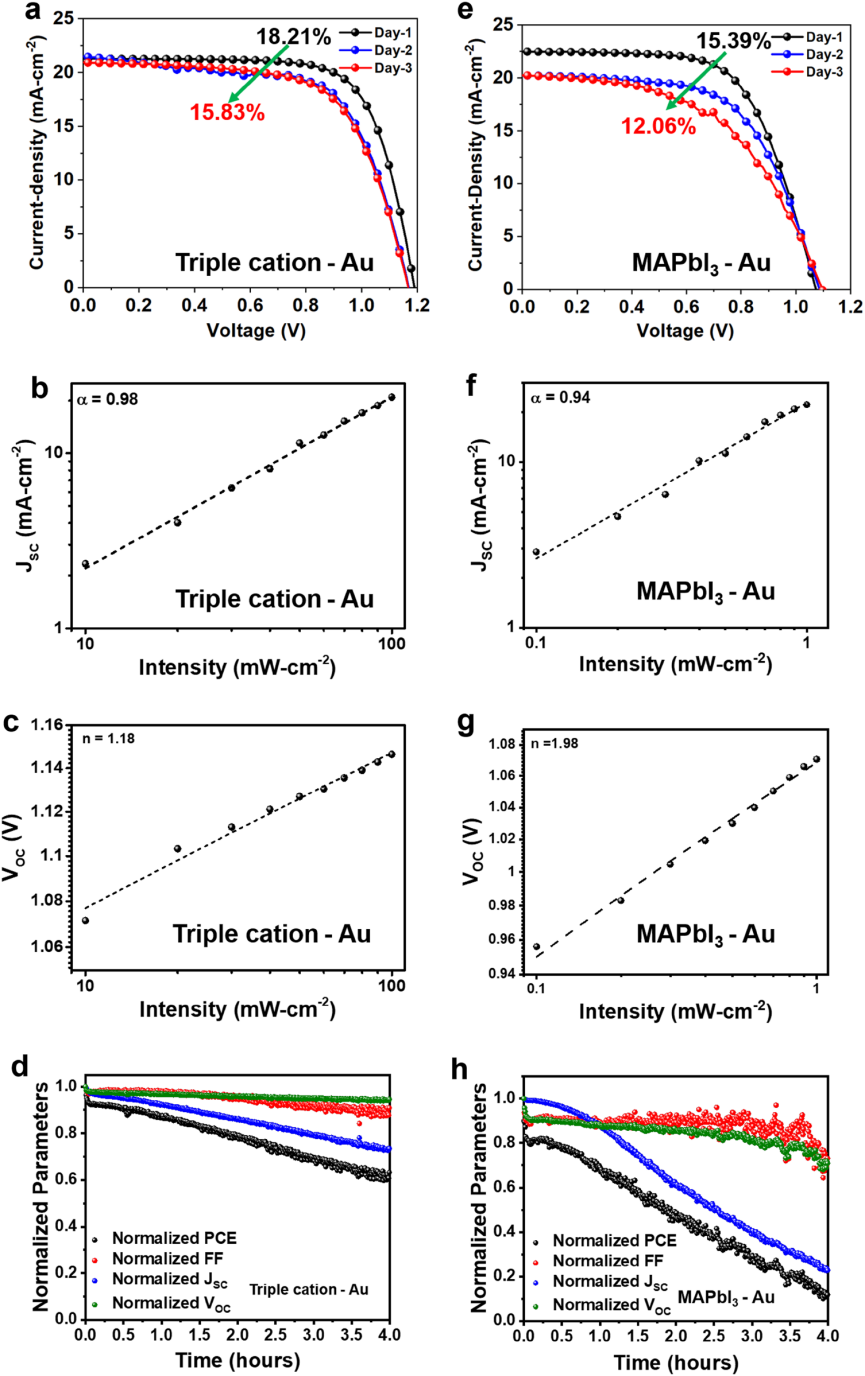
Device characterization analysis of triple cation and MAPbI_3_ PSCs with Au electrodes. For the triple cation PSC devices, shown are the: (**a**) time-dependent PCE; (**b**) *J*_SC_–*I* characteristic; (**c**) *V*_OC_–*I* characteristic; (**d**) MPPT measurements. For the MAPbI_3_ PSC devices, shown are the: (**e**) time-dependent PCE; (**f**) *J*_SC_–*I* characteristic; (**g**) *V*_OC_–*I* characteristic; (**h**) MPPT measurements. The triple-cation PSC retained ~ 60% of its initial PCE values after 4 h of testing while the MAPbI_3_ PSC degraded to ~ 10% of its initial value after the same duration.

The efficiency and time-dependent response of triple cation and MAPbI_3_ based PSCs with Au electrodes is shown in Fig. 2a–h. Specifically, Fig. 2a reveals a slower degradation in PCE (Day-1 = 18.21% to Day-3 = 15.83%) compared to the MAPbI_3_ absorber in Fig. 2e contacted with Au electrodes (Day-1 = 15.39% to Day-3 = 12.06%). Table 2 provides a summary of the time-dependent PCE device figures of merit, measured over the course of 3 days. To understand the enhanced stability of Au-based PSCs, we further examined recombination losses through light intensity-dependent *J–V* Characteristics for triple cation (Fig. 2b–d) and MAPbI_3_ absorber (Fig. 2f–h). Typically, the *J*_SC_ of the devices follows a power-law dependence with the incident light intensity *I* such that, *J*_SC_ ∝ *I*^α^; here *α* is extracted from the slope of the log–log *J*_SC_ versus *I* plot and is indicative of bimolecular recombination processes^34^. Usually, *α* close to unity indicates the presence of weak bimolecular recombination. From the data in Fig. 2b, *α* was calculated to be ~ 0.98 for the Au-contacted triple-cation absorber PSC.

**Table 2:** Time dependent PCE values of triple and MAPbI_3_ based PSCs with Au.

PSC	Day	*V*_*oc*_ (V)	*J*_*sc*_ (mA.cm^−2^)	FF (%)	PCE (%)
Triple-Au	1	1.19	21.29	71.87	18.21
Triple-Au	2	1.27	21.48	64.54	16.22
Triple-Au	3	1.16	20.93	64.78	15.83
MAPbI_3_-Au	1	1.01	22.05	60.20	15.39
MAPbI_3_-Au	2	1.08	20.23	60.67	13.33
MAPbI_3_-Au	3	1.07	20.32	55.23	12.06

Meanwhile, information about the trap-assisted recombination losses are determined using the dependence of *V*_OC_ with *I*, where ${V}_{OC}\propto \frac{nkT}{q}\mathrm{ln}\left(I\right)$. Here, $n$ is a coefficient extracted from the fitting parameters, *k* is the Boltzmann constant, *T* is the temperature in kelvin, and *q* is the elementary charge^35^. The value of the coefficient $n$ usually has a range between 1 and 2, and if $n$ ~ 1, it signifies the absence of trap-assisted recombination, while *n* approaching 2 suggests the substantial role of trap-assisted recombination in the carrier dynamics process^36^. From the fit in Fig. 2c, the value of $n$ in our triple cation PSC device was deduced to be ~ 1.18. Similar parameters extracted for the MAPbI_3_-Au contacted devices allowed us to tabulate *α* in Fig. 2f, which yielded *α* ~ 0.94, and upon comparing to the triple cation devices with α ~ 0.98 (Fig. 2b), it suggests the triple cation device has a comparatively lower bimolecular recombination than the MAPbI_3_ devices. The *n* for MAPbI_3_ PSC was deduced to be ~ 1.98 (Fig. 2g), which is indicative of higher trap-assisted recombination in the MAPbI_3_ PSCs compared to the triple-cation PSCs where *n* ~ 1.18 was deduced (Fig. 2c).

The operational stability of the best performing triple cation (Fig. 2d) and MAPbI_3_ (Fig. 2h) PSC device was deduced using the maximum power point tracking (MPPT) set-up. Here the devices were tested without any encapsulation and the device measurements were conducted at 50% relative humidity (RH) under one-sun illumination at room temperature^37^. The maximum power point was updated every 30 s by measuring the current response with a small perturbation in potential, and a *J–V* scan was taken periodically to extract the device parameters. It is noteworthy that the *V*_OC_ and *FF* of the triple cation device were quite stable over the 4-h duration of the operational test, but the *J*_SC_ decreased rapidly and retained only ~ 72% of its initial value after the end of the test (Fig. 2d). This might be due to light and oxygen-induced deterioration, which causes a decrease in photocurrent and charge extraction efficiency. The poor parameters likely arise from the formation of electron traps and trapped-states that build-up space-charge regions within the perovskite absorber layer^38^. However, these results were far better than the MAPbI_3_ PSCs where, apart from the rapid decay in *J*_SC_, a significant decrease in its *V*_OC_ and *FF* were also observed. This resulted in a precipitous drop in PCE down to ~ 10% of its initial value at the end of the test, as shown by the data in Fig. 2h.

### Material characterization

**Figure 3:**
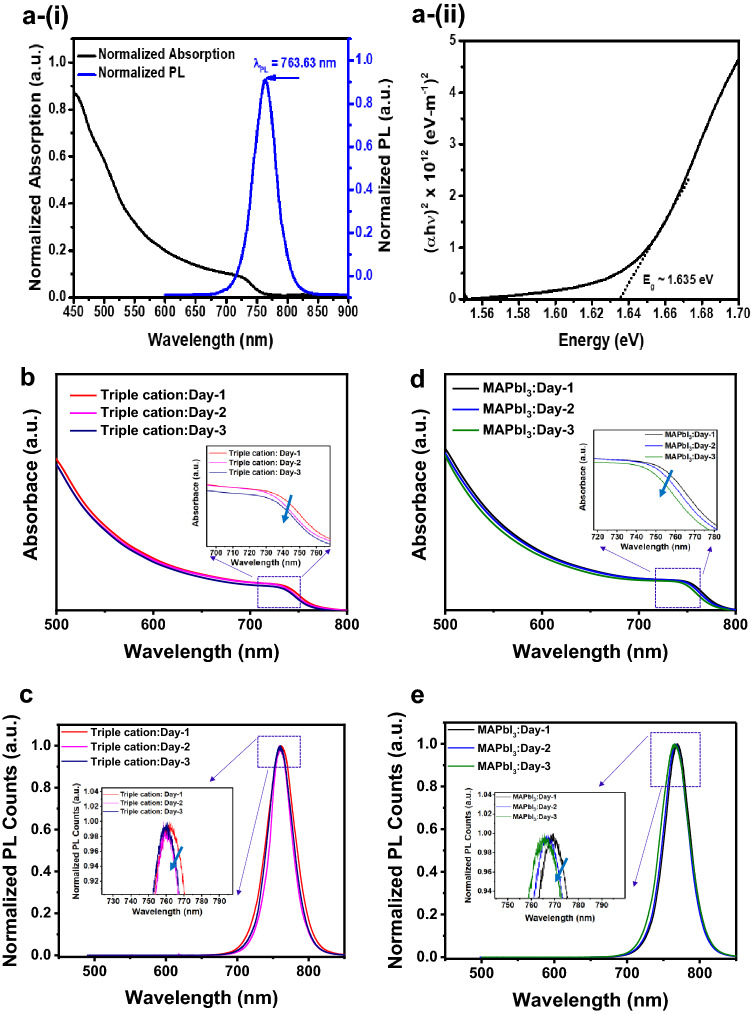
Material characterization conducted on the triple cation and MAPbI_3_ absorbers. (**a**–**i**) UV–Vis absorption spectra and steady-state PL spectra of the triple cation perovskite film on normalized scale; (**ii**) Tauc-plot for determination of the optical bandgap of the triple cation perovskite film, revealing an *E*_*g*_ ~ 1.63 eV. For the triple cation absorber, evolution of (**b**) absorbance spectra and (**c**) PL spectra with ambient exposure from day 1 to day 3. Insets in (**b**,**c**) represent the expanded scale spectra. For the MAPbI_3_ absorber, evolution of (**d**) absorbance spectra and (**e**) PL spectra with ambient exposure from day 1 to day 3. Insets in (**d**,**e**) represent the expanded scale spectra. The ambient exposure conditions for the films were ~ 22 °C and ~ 50% RH.

To shed insights on the role of the electrode material for influencing stability of the PSCs, various material characterization probes were used. Firstly, we examined the optical properties of our synthesized triple cation perovskite films by UV–Vis and PL, as shown in Fig. 3a–i. Here the triple cation film showed a decent optical absorption in the visible regime and the steady-state PL emission peak occurred at ~ 763.63 nm, which is in close agreement with earlier reports for the same composition^13^. The corresponding optical bandgap *E*_g_ of the triple cation film in Fig. 3a-(ii) was calculated using the UV–Vis optical absorption spectra fit to the Tauc equation, i.e. $${\left(\alpha (1)hv\right)}^{n1}=A(hv-{E}_{g}).$$ Here, *α(1)* is the absorption coefficient calculated as $$2.303\times \frac{absorbance}{thickness of the film}$$, where absorbance values were obtained from the UV–Vis absorption spectra and the thickness of the perovskite film was assumed to be ~ 350 nm based on previously used spin-coating parameters from prior fabrication runs, *hν* is the energy of the incident photon (with *h* being Planck’s constant = 6.626 $$\times $$ 10^–34^ J s and *ν* is the frequency) and *A* is a constant; *n1* can have two different values, either 2 for direct or 0.5 for indirect bandgap transitions. Since organic–inorganic metal halide perovskites are direct bandgap semiconductors in the bulk, we assumed *n1* = 2. The intercept on the *x*-axis of the Tauc-plot [(*α*(*1*)*hν*)^2^ versus *hν*] yields an approximate value of *E*_g_ for the material. As shown in Fig. 3a-(ii), the extrapolation of the (*α*(*1*)*hν*)^2^ curve results in *E*_g_ ~ 1.635 eV for the triple cation perovskite film, consistent with prior reports. For the more commonly used MAPbI_3_ perovskite absorber, *E*_g_ ~ 1.63 from prior work^39^. At the same time, the optical properties, namely absorption and PL spectra, for the double cation (MA/FA) absorbers we synthesized are shown in Figures S1a,b, respectively, which reveals the increase in absorption for the double cation compared to MAPbI_3._ The PL data indicates a red-shift in the emission peak of the double cation absorber to ~ 779 nm, compared to ~ 768 nm for the MAPbI_3_.

In order to analyze the intrinsic stability of the synthesized triple cation and MAPbI_3_ perovskite films in our specific laboratory conditions, we measured their optical properties using UV–Vis and PL over the course of several days, as shown by the data in Fig. 3b–e. By gauging the intrinsic stability of the absorbers, it helped us to decouple the degradation induced via the electrodes. For the triple cation film (Fig. 3b), there is a slightly blue-shifted absorption band edge (as reflected in the inset on a magnified scale), compared to the MAPbI_3_ perovskite (Fig. 3d), which is likely due to quantum confinement effects associated with the smaller bromine ions compared to the iodine as the halide in the given structures. Bromine halide-based perovskites are known for their higher bandgap compared to iodine-based perovskites^40^. Both films were further subject to ambient exposure at ~ 22 °C and ~ 50% RH to study their storage stability, by acquiring the optical absorption characteristics with exposure. Both films showed very slow deterioration in optical absorption, which is seen by a slight blue shifting of the spectra from Day-1 to Day-3 in Fig. 3b–d, but the absorbance of the triple cation film decayed at a slower rate compared to the MAPbI_3_. This confirms the triple cation perovskite is more stable when exposed to ambient conditions compared to the MAPbI_3_ film^41^. The equivalent PL data is shown in Fig. 3c,e for the triple cation and MAPbI_3_ films, respectively. Here, the exposed films reveal a similar trend of a slightly blue-shifted peak location (as evident from the magnified plots in the respective insets) for both the triple cation and the MAPbI_3_ films, upon exposure to ambient conditions over a course of 3 days. The time-dependent optical absorption and PL data reveal that the degradation is not significantly accelerated through the mere exposure of the absorber to ambient conditions.

**Figure 4:**
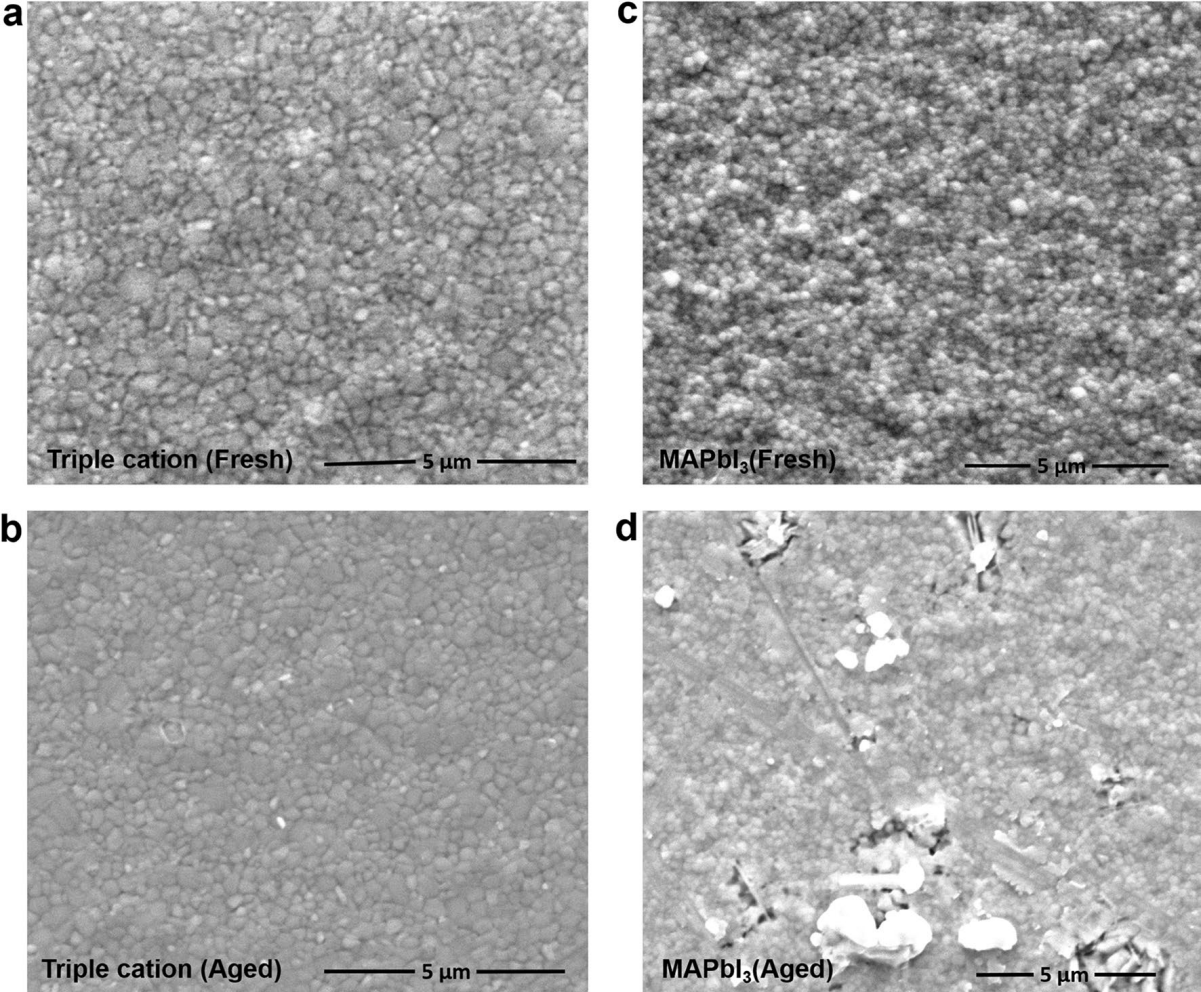
SEM images of (**a**) fresh and (**b**) aged triple cation perovskite absorber on FTO/TiO_2_. The exposure to ambient conditions included 10 days of storage in a petri dish/at 25 °C and RH ~ 30 to 40%. SEM images of (**c**) fresh and (**d**) aged MAPbI_3_ perovskite absorber on FTO/TiO_2_. The fresh and aged triple cation appeared to be more dense compared to the MAPbI_3_ whose porosity increased with aging leading to potential avenues for nucleating degradation mechanisms.

In order to understand the morphological changes with exposure to ambient conditions, SEM analysis was conducted on the fresh and aged perovskite absorbers and the resulting images are shown in Fig. 4a–d. Imaging on the fresh triple cation absorber (Fig. 4a) revealed a smooth and relatively dense, pinhole-free morphology and the image in Fig. 4b for the aged triple cation-based absorber films still showed a fairly smooth surface morphology despite being exposed to ambient conditions noted previously. Similarly, the fresh MAPbI_3_ (Fig. 4c) based absorber films showed a more dramatic change in surface morphology after exposure (Fig. 4d) with more defects and porosity^42,43^. This data are helpful to explain the device behaviour, since it is well known that crystallinity and structural attributes of the absorber are crucial for the intrinsic and extrinsic stability of the perovskite films, where the main degradation process starts at defect sites such as grain boundaries given their increased porosity^44^. At the same time, SEM imaging of the double cation (MA/FA) absorber compared to the MAPbI_3_ are shown in Figure S2a,b. These results reveal the denser film morphology of the double cation in Figure S2b, compared to the MAPbI_3_ in Figure S2a.

**Figure 5:**
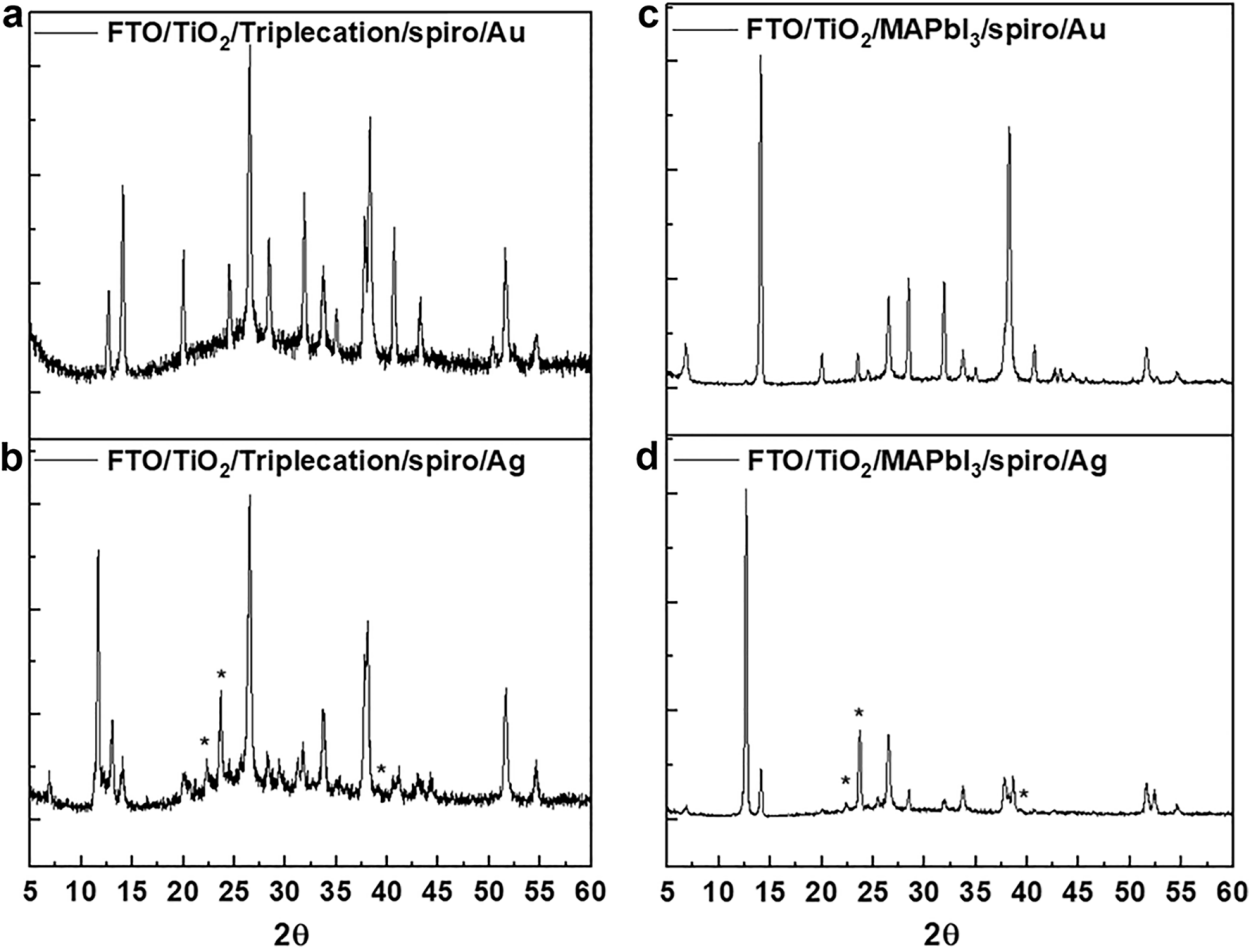
XRD of aged PSCs stored in ambient condition over 10 days in a petri dish without encapsulation at 25 °C and RH ~ 30 to 40%. XRD spectra of: (**a**) triple cation with Au; (**b**) triple cation with Ag; (**c**) MAPbI_3_ with Au; (**d**) MAPbI_3_ with Ag. Asterisks in (**b**) and (**d**) indicate the diffractions at 2θ = 22.3°, 23.8°, and 39.2° corresponding to the (100), (002), and (111) peaks of β-AgI, respectively. These peaks indicate the rapid formation of AgI in Ag based PSCs, while in (**a**,**c**), the formation of AuI is almost negligible since the characteristic AuI diffraction peaks were absent from our spectra, as noted in the Supporting Information of Ref.^30^.

Next, we measured XRD for n-i-p PSCs with both Au and Ag electrodes. We used the aged PSCs that were stored at ambient conditions noted above without encapsulation for 10 days in a petri dish without encapsulation at 25 °C and RH ~ 30-0% to understand the role of the electrode layer in determining the stability and ensuing performance of the cell. Cacovich et al*.* also reported that long-term light exposure of PSCs causes Au and I migration from the metal contact and the perovskite layer, respectively^28^. However, one important issue concerning the degradation stemming from Au electrodes in PSCs through ion migration, published by Besleaga et al*.*, is the strong dependence of stability on the absence/presence of pinholes in the perovskite thin films^26^. There appears to be little discussion in the literature on the role of Au-electrodes for explaining degradation mechanisms in PSCs. However, it is clear that degradation in PSCs associated with the electrodes is an important issue to address in deciding the stability of PSCs. Thus, it is necessary to address this critical issue to advance PSCs in terms of their long-term stability. The XRD spectra of triple-cation and MAPbI_3_ based PSCs with Au and Ag electrodes are shown in Fig. 5a,b and c,d. Since we stored our PSCs at ambient conditions (which includes exposure to moisture and UV light), multiple degradation pathways maybe at play here. This seems to be the case since multiple unknown phases are evident in the XRD spectra of the triple cation and MAPbI_3_ based PSCs with Au. Unlike AgI, the formation of AuI can occur only at elevated temperatures over 170 °C, and so we expect in our case the formation of AuI to be quite minimal. Additionally, we did not find any notable AuI formation in Fig. 5a,b, which is consistent with observations made by Tarasov et al*.* (please see their supporting information)^31^. However, a vigorous reaction occurs between I_2_ and metallic lead (reaction with a mixture of I_2_ and MAI) often leads to the formation of reactive polyiodide melts (RPMs) even at room temperature. The RPMs contain ionic species, such as MA^+^ that would favor the formation of complex phases of an ionic origin, instead of the less stable binary phases, such as AuI. Thus, the formation of RPMs during UV-irradiation of the MAPI/Au mixture and the subsequent reaction of RPMs with metallic Au is likely to be the main reason and the key origin of the formation of unknown phases in the XRD (Fig. 5a,b). This was further supported by Pistor et al*.*^45^ who report on MAPbI_3_ degradation using a 532 nm laser in a Raman spectrometer through the formation of RPMs. On the other hand, the Ag-based PSCs showed clear diffractions typical of AgI, in both triple cation and MAPbI_3_ based PSCs. As shown in Fig. 5b,d, the peaks labelled with the star are indexed at diffraction angles of 2θ = 22.3°, 23.8°, and 39.2° corresponding to the (100), (002), and (111) peaks of β-AgI, respectively, which are in agreement with previous reports^33^. Incidentally, XRD results on the double cation (MA/FA) absorber compared to the MAPbI_3_ are shown in Figure S3.

**Figure 6:**
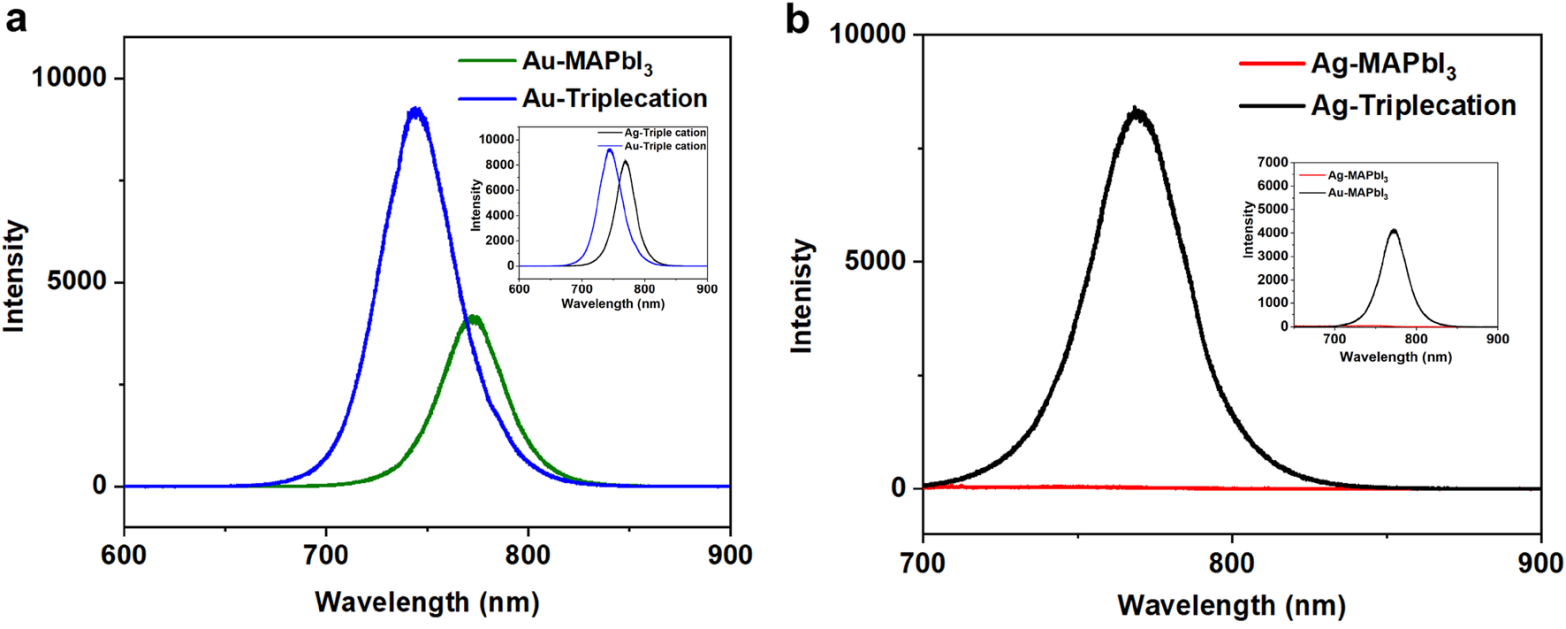
The PL spectra acquired using a 532 nm excitation laser, as it probed the absorber in situ within the solar cell stack in the n-i-p device architecture directly. (**a**) PL spectra of aged triple cation and MAPbI_3_ based PSCs, where the cells were stored in ambient conditions for ~ 10 days in a petri dish without encapsulation at 25 °C and RH ~ 30 to 40% with Au electrodes. Inset represents peak shift between the Ag and Au based triple cation based perovskite. (**b**) PL spectra of aged triple cation and MAPbI_3_ based PSCs with Ag electrodes. Inset represents the peak-shift between the Ag and Au-contacted MAPbI_3_ within the solar cell stack. As shown in (**a**), PSCs with Au electrodes showed notable PL emission with a clearly visible peak maxima, which validates that both absorbers in Au-based PSCs are less vulnerable to degradation even with the formation of AuI. On the other hand, MAPbI_3_ based PSCs in (**b**) showed a negligible PL peak over the same triple cation-based PSCs contacted with the Ag, which further supports the possible rapid degradation of MAPbI_3_ based PSCs with Ag electrodes over the triple cation-based PSCs since the absorber in the former appears to be optically degraded through these in situ PL measurements to access the absorber layers for the degradation study mediated via the Ag and Au collector electrodes.

As an additional material probe to unveil the metal-collector associated degradation in the PSCs, here we illustrate the novel use of PL to access key features of the perovskite absorber in situ, while it was in contact with the various layers within the n-i-p solar cell stack. The laser within the PL system used for the excitation source was focused on top of the Spiro-OMeTAD layer in the lateral vicinity of the opaque metal electrodes, and penetrated through the Spiro-OMeTAD layer to access the absorber, within in the solar cell stack of the fabricated and aged cells. The PL spectra of the triple cation and MAPbI_3_ based PSCs with Au and Ag electrodes was measured and the corresponding data are shown in Fig. 6a,b, respectively. The PSCs with Au electrodes showed a notable PL emission for both absorbers with an observable maxima, as shown in Fig. 6a, providing direct evidence that the Au-based PSCs for both absorbers lead to optically active absorbers while residing within the stack, and they are less vulnerable to degradation or the formation of AuI. On the other hand, MAPbI_3_ based PSCs contacted with Ag showed negligible PL emission, while the triple cation-based PSC showed an optically active peak, confirmed in Fig. 6b, which further supports the possible rapid degradation of MAPbI_3_ based PSCs with Ag compared to the triple cation-based PSCs with the same Ag-electrode. The quenching in the PL peak for the Ag-contacted MAPbI_3_ provided direct evidence of the Ag corrupting the optical properties of the absorber through the formation of AgI. In addition, we also examined the PL spectra of triple cation and MAPbI_3_ based PSCs with the two Au and Ag electrodes, where the data are displayed in the insets of Fig. 6a,b, respectively. As shown in the inset of Fig. 6a, the triple cation PSC with Au showed higher PL intensity compared to the triple cation contacted with Ag, which is well aligned with their respective PCEs from the data presented in Fig. 1. On the other hand, in the inset in Fig. 6b, the near absent PL peak for MAPbI_3_ PSC with Ag electrodes compared to the Au PSC, supports our PCE gathered from the device data in Fig. 1, while also confirming the formation of AgI as the plausible reason for the PL quenching.

## Conclusions

In summary, we successfully fabricated triple cation and MAPbI_3_ based PSCs with both Ag and Au metal collector electrodes. The optimized PCE of triple cation and MAPbI_3_-based PSCs with Au electrode is 18.21% and15.39%, respectively. On the other hand, optimized PCE of triple cation and MAPbI_3_ based PSCs with Ag electrodes were 16.44% and 3.02%, respectively. Notably, triple cation and MAPbI_3_-based PSCs with Au electrodes showed an improved PCE compared to Ag-based PSCs. The high PCE and relatively better stability of Au-based PSCs is attributable to the slow-paced degradation of absorbers in Au-based PSCs and the formation of RPMs. The rapid degradation of absorbers and immediate formation of AgI in triple cation and MAPbI_3_ based PSCs with Ag electrodes is the major reason for the poor stability and lower PCE. Additionally, the relatively better stability of the triple cation-based PSCs over MAPbI_3_-based PSCs with Ag electrodes is due to the intrinsic stability of the triple cation absorber. Overall, this work reveals that Au is an appropriate choice for realizing stable PSCs to enhance device longevity through device operational stability and environmental storage tests conducted, highlighting the importance of electrodes in contributing toward the overall stability of PSCs.

## Materials and methods

### Materials

Lead (II) iodide (PbI_2_, 99.99%) was purchased from TCI America, while *N*,*N* dimethylformamide (DMF), dimethylsulfoxide (DMSO), anhydrous ethanol, toluene, chlorobenzene, methylammonium bromide (MABr), lead (II) bromide (PbBr_2_), caesium iodide (CsI), methylammonium iodide (MAI, 99.9%), and formamidinium iodide (FAI, > 98%), tris(2-(1H-pyrazol-1-yl)-4-tert-butylpyridine)cobalt(III) tris-(bis(trifluoromethyl sulfonyl)imide) (FK209). were purchased from Sigma-Aldrich. The 2,2′,7,7′-tetrakis-(*N*,*N*-di-p-methoxyphenylamine)-9,9′-spirobifluorene (Spiro-OMeTAD) was obtained from Lumtec. Finally, fluorine-doped tin oxide (FTO) coated glass substrates (dimensions: 25 mm × 25 mm × 2.2 mm; sheet resistance: 7–8 Ω sq^−1^), were purchased from Zhuai Kaivo, China. All chemicals were used as received.

### Substrate etching and cleaning

The cleaning procedure of the pre-etched FTO coated glass substrates with area of 25 × 15 mm, encompassed the following steps. First, we cleaned the substrates using water with Hellmanex, deionized (DI) water, ethanol, and isopropanol (IPA) consecutively for 20 min. under sonication. The cleaned substrates then were dried using N_2_ and finally, the substrates were placed in a convection oven at 70 °C for 30 min.

### Device fabrication

#### Triple-cation (Cs_0.05_FA_0.79_MA_0.16_PbI_2.45_Br_0.55_) perovskite solar cells

For the PSC device fabrication, cleaned glass/FTO substrates were treated with a UV-ozone vapor for 20 min. just before the deposition of the subsequent films. The UV-ozone treated FTO substrates were then spin-coated with a 0.15 M solution of titanium diisopropoxide bis(acetylacetonate) in ethanol at 3000 rpm for 20 s to deposit a compact titanium dioxide (c-TiO_2_) electron transport layer (ETL), which is then annealed at 510 °C for 30 min. For preparation of triple-cation perovskite ink, first, FAI (1 M), MABr (0.2 M), PbI_2_ (1.1 M) and PbBr_2_ (0.2 M) were dissolved in fresh anhydrous DMF:DMSO (4:1 volume ratio), the solution was stirred at 65 °C for 2 h. Then, a separate solution of CsI (1.5 M) in 1 mL of DMSO was prepared and 50 µL of this CsI solution was added per mL to the above stock solution to give the desired solution composition of Cs_0.05_FA_0.79_MA_0.16_PbI_2.45_Br_0.55_. The solution was further heated to 65 °C for 2 h. Triple-cation perovskite films were then deposited using the precursor ink on glass/FTO/C-TiO_2_ substrates via the one-step solvent engineering deposition method. The spinning conditions were as follows: (1) 1000 rpm for 8 s and (2) 5100 rpm for 20 s with 100 μL of chlorobenzene antisolvent dripped on the film around 5 s before the end, with the film immediately turning translucent brown. Films were then transferred to a hotplate for annealing at 125 °C for 20 min. After the deposition of the perovskites layer, the hole transport layer (HTL) is then deposited. To prepare the solution for the HTL, spiro-OMeTAD (86 mg mL^−1^ in chlorobenzene) was doped with three solutions, i.e. 34 µL of TBP, 20 µL of Li-TFSI and 11 µL FK209. For the latter two solutions, Li-TFSI and FK209, a mother solution of 500 mg mL^−1^ and 300 mg mL^−1^, respectively, in acetonitrile was used. This doped spiro-OMeTAD solution was then spin-coated onto the perovskite layer at 4000 rpm for 20 s. Lastly, an ~ 80 nm thick metal electrode layer (Ag or Au), was deposited using e-beam evaporation at a vacuum pressure of ~ 10^–5^ Torr, with an active device area of ~ 0.2 cm^2^.

#### MAPbI_3_ perovskite solar cells

For preparation of MAPbI_3_ perovskite ink, first, MAI(1.5 M) and PbI_2_ (1.5 M) were dissolved in fresh anhydrous DMF:DMSO (4:1 volume ratio) and the solution was stirred at 65 °C for 2 h. All other steps of device fabrication with MAPbI_3_ perovskite photoabsorber were the same as in the case of the triple cation PSCs with the exception that for the MAPbI_3_ PSCs, toluene was used as the antisolvent in place of chlorobenzene. Thus, 100 μL of toluene antisolvent was dripped on the film around 5 s before the spin coating process ends.

In measurements where perovskite layers were prepared for optical characterization, perovskite films were simply deposited using the above protocol on cleaned, UV-ozone treated FTO glass substrates. All processing related with the perovskite and HTL was conducted in an Ar-filled glovebox with H_2_O and O_2_ levels of < 0.1 ppm.

### Optical characterization

Agilent CARY 5000 spectrophotometer was used to conduct the Ultraviolet–visible (UV–Vis) absorption spectroscopy of the synthesized films. Steady-state PL spectra were gathered using a ~ 532 nm LASER source equipped with LabRAM HR Evolution spectrometer from HORIBA Scientific.

### Device characterization

The electrical characteristics of the fabricated solar cells were measured using the Oriel LSH-7320 LED solar simulator which is connected to a source meter unit from Ossila (Model: X200). The measurement was done under one-sun of optical illumination, i.e. 100 mW-cm^−2^. The PVM-396 reference cell from PV Measurements Inc. certified by the US National Renewable Energy Laboratory (NREL) was used for calibration.
